# Distribution of CRISPR in *Escherichia coli* Isolated from Bulk Tank Milk and Its Potential Relationship with Virulence

**DOI:** 10.3390/ani12040503

**Published:** 2022-02-17

**Authors:** Hyo-Jung Kang, Young-Ju Lee

**Affiliations:** College of Veterinary Medicine and Zoonoses Research Institute, Kyungpook National University, Daegu 41566, Korea; sa01083@knu.ac.kr

**Keywords:** *E. coli*, virulence gene, phylogeny, CRISPR array, bulk tank milk

## Abstract

**Simple Summary:**

In the dairy farms of many different countries, *E. coli* is one of the most common causes of mastitis. It is defined as mammary pathogenic *E. coli*, and is known to cause opportunistic infections by possessing diverse virulence factors. Therefore, the purpose of this study was to investigate the virulence potential of *E. coli* isolates from bulk tank milk in Korea, and observe its association with clustered regularly interspaced short palindromic repeat (CRISPR) arrays. The results showed that out of 183 isolates, 164 (89.6%) possessed one or more of 18 virulence genes, and belonged to phylogenetic groups B1 (64.0%), A (20.1%), D (8.5%), and C (7.3%). CRISPR arrays of *E. coli* are classified as either CRISPR I-E (CRISPR 1 and 2) or CRISPR I-F (CRISPR 3 and 4). In this study, only CRISPR 1 (95.7%) and 2 (74.4%) were detected. Among the eight protospacers matching plasmids and phages, three were associated with gene regulation, and one was associated with virulence. Moreover, the different virulence genes showed significantly different patterns of CRISPR distribution and CRISPR sequence-types. This result implies that CRISPR loci may be associated with gene regulation and pathogenicity in *E. coli,* and that the CRISPR sequence-typing approach can help to clarify and trace virulence potential, even though the *E. coli* isolates were from normal bulk tank milk.

**Abstract:**

*Escherichia coli* is one of the most common causes of mastitis on dairy farms around the world, but its clinical severity is determined by a combination of virulence factors. Recently, clustered regularly interspaced short palindromic repeat (CRISPR) arrays have been reported as a novel typing method because of their usefulness in discriminating pathogenic bacterial isolates. Therefore, this study aimed to investigate the virulence potential of *E. coli* isolated from bulk tank milk, not from mastitis, and to analyze its pathogenic characterization using the CRISPR typing method. In total, 164 (89.6%) out of 183 *E. coli* isolated from the bulk tank milk of 290 farms carried one or more of eighteen virulence genes. The most prevalent virulence gene was *fimH* (80.9%), followed by *iss* (38.3%), *traT* (26.8%), *ompT* (25.7%), *afa/draBC* (24.0%), and *univcnf* (21.9%). Moreover, the phylogenetic group with the highest prevalence was B1 (64.0%), followed by A (20.1%), D (8.5%), and C (7.3%) (*p* < 0.05). Among the four CRISPR loci, only two, CRISPR 1 and CRISPR 2, were found. Interestingly, the distribution of CRISPR 1 was significantly higher in groups A and B1 compared to that of CRISPR 2 (*p* < 0.05), but there were no significant differences in groups C and D. The prevalence of CRISPR 1 by virulence gene ranged from 91.8% to 100%, whereas that of CRISPR 2 ranged from 57.5% to 93.9%. The distribution of CRISPR 1 was significantly higher in *fimH*, *ompT*, *afa/draBC*, and *univcnf* genes than that of CRISPR 2 (*p* < 0.05). The most prevalent *E. coli* sequence types (EST) among 26 ESTs was EST 22 (45.1%), followed by EST 4 (23.2%), EST 16 (20.1%), EST 25 (19.5%), and EST 24 (18.3%). Interestingly, four genes, *fimH*, *ompT*, *afa/draBC*, and *univcnf*, had a significantly higher prevalence in both EST 4 and EST 22 (*p* < 0.05). Among the seven protospacers derived from CRISPR 1, protospacer 163 had the highest prevalence (20.4%), and it only existed in EST 4 and EST 22. This study suggests that the CRISPR sequence-typing approach can help to clarify and trace virulence potential, although the *E. coli* isolates were from normal bulk tank milk and not from mastitis.

## 1. Introduction

*Escherichia coli* is one of the most common Gram-negative bacteria residing in the intestines of animals in an anaerobic and facultative manner [1]; however, it is also one of the most common causes of mastitis on dairy farms [2]. Generally, *E. coli* mastitis results in a subclinical pathology caused by an environmental opportunistic infection. However, the presence of diverse virulence properties associated with extraintestinal pathogenesis, such as adhesins, toxins, capsule synthesis, siderophores, invasins, and serum survival, is reported to be crucial for the colonization of the mammary glands via increased bacterial survival and tissue damage [3,4]. In particular, Aslam et al. (2021) [5] reported that the presence of various virulence genes in extraintestinal pathogenic *E. coli* (ExPEC) contributed to the rise in mammary pathogenic *E. coli* (MPEC).

Moreover, the virulence potential necessary to cause an infection of the mammary glands is determined by a combination of factors, not the presence of a single factor [6]. Hence, phylogenetic analysis is important because it enriches the understanding of the classification, and determines the virulence of pathogenic *E. coli* [7,8]. *E. coli* is derived from different phylogenetic groups A, B1, B2, C, D, E, and F [9], and the majority of strains responsible for ExPEC, such as uropathogenic *E. coli* (UPEC), newborn meningitis-associated *E. coli*, and avian pathogenic *E. coli*, belong to groups B2 and D [10,11]. However, even though MPEC can cause infections outside of the gastrointestinal system, both MPEC and bovine commensals belong to phylogroups A and B1, because MPEC may be recruited from the normal intestinal commensal microbiota [12,13,14]. 

Clustered regularly interspaced short palindromic repeat (CRISPR) arrays are a bacterial adaptive immune system that neutralizes invading phages and plasmids by cutting foreign DNA at specific locations. It consists of various spacers, which are short sequences between each repeat [15,16,17]. A protospacer, which is a short external sequence at a specific location, is inserted as a spacer in the CRISPR loci of bacteria during an infection [18]. Recently, CRISPR arrays have been applied as a novel typing method for isolates because they are useful in discriminating the pathogenicity of *Salmonella* [19], *E. coli* [20,21,22,23,24,25], and *Pseudomonas aeruginosa* [26]. This study aimed to investigate the virulence potential of *E. coli* isolated from bulk tank milk, not from mastitis, and to analyze the pathogenic characterization of *E. coli* using the CRISPR typing method.

## 2. Materials and Methods

### 2.1. Bacterial Strains

Each 50 mL of bulk tank milk was aseptically collected, from 290 farms operated by three dairy companies, and tested in accordance with the standard microbiological protocols published by the Ministry of Food and Drug Safety (2018) [27]. Approximately 1 mL of each bulk tank milk sample was inoculated into 9 mL of modified *Escherichia coli* broth (Merck, Darmstadt, Germany), and these were incubated at 37 °C for 24 h. A loopful of enriched mEC was streaked onto MacConkey agar (BD Bioscience), and incubated at 37 °C for 24 h. Three typical colonies selected from each sample were confirmed by PCR, as described previously [28]. If isolates of the same origin showed the same antimicrobial susceptibility patterns, only one isolate was randomly chosen, and a total of 183 *E. coli* were included in this study.

### 2.2. Detection of Virulence Genes

A total of 33 virulence genes associated with ExPEC (afa/draBC, bmaE, cdt, cdtB, cnf1, cvaC, fimH, focG, fyuA, hlyA, ibeA, ireA, iroN_E. coli_, iss, iutA, kpsMT K1, kpsMT2, kpsMT3, kpsMT K5, nfaE, ompT, PAI, papAH, papC, papEF, papG allele 1, papG allele 2, papG allele 3, rfc, sfa/focDE, sfaS, traT, and univcnf) were screened by PCR, as described previously [29].

### 2.3. Phylogenetic Groups

Phylogenetic grouping was accomplished by a multiplex PCR-based method using *chuA, yjaA, TSPE4.C2*, *arpA*, and *trpA* genes, and the bacteria were assigned into groups A, B1, B2, C, D, E, F, and clade I, as described previously [9].

### 2.4. CRISPR Locus Sequence Typing and Spacer Analysis

Four CRISPR loci were screened by PCR, as described previously [22]. The PCR products were purified using a QIAquick PCR purification kit (Qiagen, Hilden, Germany), and sequenced using an automatic sequencer (Cosmogenetech, Seoul, Korea). Sequences were analyzed by CRISPRFinder (https://crispr.i2bc.paris-saclay.fr/Server/, accessed on 31 August 2021), as described by Grissa et al. (2007) [30], and only spacers were obtained for this study. All *E. coli* were categorized into *E. coli* sequence types (ESTs) based on their spacer distributions, numbered arbitrarily. The name and full sequences of all spacers are listed in Supplementary Table S1. CRISPRTarget (http://crispr.otago.ac.nz/CRISPRTarget/crispr_analysis.html, accessed on 31 August 2021) with a cut-off value of 29, and nucleotide BLAST (https://blast.ncbi.nlm.nih.gov/Blast.cgi, accessed on 10 September 2021) were used to detect protospacers derived from phages or plasmids [31].

### 2.5. Statistical Analysis

Analysis via the Statistical Package for the Social Sciences version 25 (IBM SPSS Statistics for Windows, Armonk, NY, USA) was used in this study. Pearson’s chi-square test and Fisher’s exact test with Bonferroni correction were conducted to analyze the differences associated with the distribution of virulence genes, phylogenetic groups, and ESTs. A *p*-value < 0.05 was considered statistically significant.

## 3. Results

### 3.1. Distribution of Virulence Genes

The distribution of 33 virulence genes associated with ExPEC in *E. coli* from bulk tank milk is presented in Table 1. A total of 164 (89.6%) out of 183 *E. coli* isolated from bulk tank milk carried one or more of eighteen virulence genes. The most prevalent virulence gene was *fimH* (80.9%), followed by *iss* (38.3%), *traT* (26.8%), *ompT* (25.7%), *afa/draBC* (24.0%), and *univcnf* (21.9%). Interestingly, both *iss* and *traT* had the significantly highest prevalence in *E. coli* from company A, and *fimH* had a significantly higher prevalence in *E. coli* from companies A and B (*p* < 0.05). Although *kpsMTK5* had a low prevalence (7.1%) in *E. coli*, this gene also showed a significant difference among the companies (*p* < 0.05).

### 3.2. Distribution of Phylogenetic Groups and CRISPR Loci

The distribution of phylogenetic groups and CRISPR loci in 164 *E. coli* isolates with some virulence genes is presented in Table 2. All isolates were assigned into four phylogenetic groups: A, B1, C, and D. The phylogenetic group with the significantly highest prevalence was B1 (64.0%), followed by A (20.1%), D (8.5%), and C (7.3%). Although the distribution of groups B1 and C was not significantly different by company, that of groups A and D showed significant differences between companies (*p* < 0.05). Among the four CRISPR loci examined, only two, CRISPR 1 and CRISPR 2, were found. However, the prevalence of CRISPR 1 (95.7%) was significantly higher than that of CRISPR 2 (74.4%) (*p* < 0.05). On the other hand, no significant differences between the companies were observed.

### 3.3. Distribution of CRISPR 1 and CRISPR 2 by Phylogenetic Group

**Figure 1:**
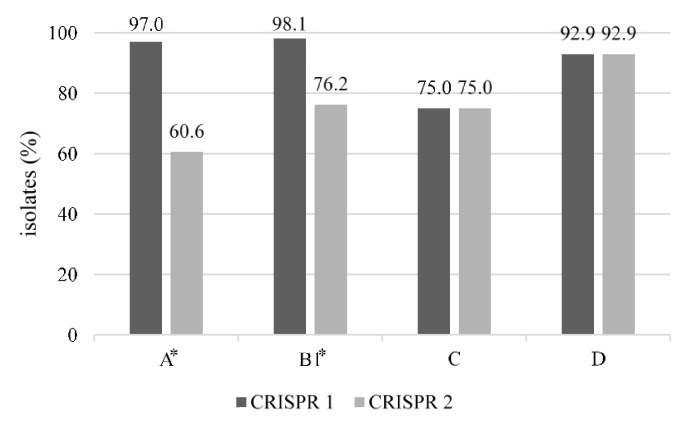
Distribution of CRISPR 1 and CRISPR 2 by phylogenetic group in 164 *E. coli* possessing virulence genes, isolated from bulk tank milk. The asterisk indicates that there were significant differences between CRISPR 1 and CRISPR 2 (*p* < 0.05).

The association of CRISPR 1 and CRISPR 2 with phylogenetic groups of *E. coli* is shown in Figure 1. The prevalence of CRISPR 1 by phylogenetic groups ranged from 75.0% to 98.1%, whereas that of CRISPR 2 ranged from 60.6% to 92.9%. Interestingly, the distribution of CRISPR 1 and CRISPR 2 in the phylogenetic groups showed significant differences. The distribution of CRISPR 1 was significantly higher in groups A and B1 than that of CRISPR 2 (*p* < 0.05). The prevalence of CRISPR 1 and CRISPR 2 showed no significant differences in groups C and D.

### 3.4. Distribution of CRISPR 1 and CRISPR 2 by Virulence Genes

**Figure 2:**
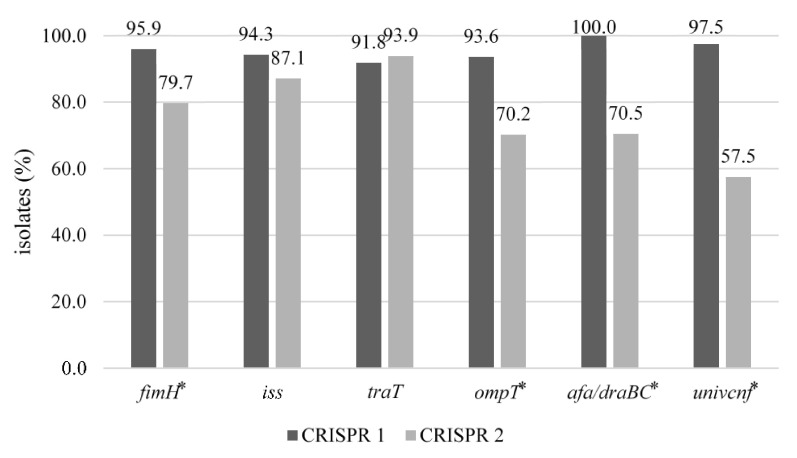
Distribution of CRISPR1 and CRISPR2 by virulence gene in 164 *E. coli* possessing virulence genes, isolated from bulk tank milk. The asterisk indicates that there were significant differences between CRISPR 1 and CRISPR 2 (*p* < 0.05).

The association of CRISPR 1 and CRISPR 2 with six common virulence genes of *E. coli* is shown in Figure 2. The prevalence of CRISPR 1 by virulence gene ranged from 91.8% to 100%, whereas that of CRISPR 2 ranged from 57.5% to 93.9%. The distribution of CRISPR loci also showed differences by virulence gene. The distribution of CRISPR 1 was significantly higher in *fimH*, *ompT*, *afa/draBC*, and *univcnf* genes than that of CRISPR 2 (*p* < 0.05). Moreover, *iss* and *traT* genes showed equally high distributions, and no significant differences between CRISPR 1 and CRISPR 2.

### 3.5. CRISPR-Based Typing of Virulence Gene-Carrying Isolates

The distribution of ESTs by six common virulence genes of *E. coli* is presented in Table 3. A total of 26 ESTs were assigned based on the distribution of spacers from CRISPR 1 and CRISPR 2. The most prevalent EST was EST 22 (45.1%), followed by EST 4 (23.2%), EST 16 (20.1%), EST 25 (19.5%), and EST 24 (18.3%). Interestingly, four genes, *fimH*, *ompT*, *afa/draBC*, and *univcnf*, had a significantly higher prevalence in both EST 4 and EST 22, while *iss* and *traT* genes had a significantly lower prevalence than the other four genes in both EST 4 and EST 22 (*p* < 0.05).

### 3.6. Protospacer Match from Spacer Sequences

The protospacers matching plasmids and phages, and sequences are presented in Table 4. Seven and one protospacers were found in CRISPR 1 and CRISPR 2, respectively. Interestingly, protospacer 163, which is associated with virulence due to the tail-associated lysozyme of bacteriophage, had the highest prevalence (20.4%), and it only existed in EST 4 and EST22 (Supplementary Figure S1). Moreover, other protospacers were concerned with the protection of bacteria against the host immune system, such as toll/interleukin-1 receptor domain-containing protein (0.6%), or gene regulation, such as DNA-binding protein (18.5%), DNA-cytosine methyltransferase (7.0%), and *darB,* helicase (0.6%). However, protospacer 117 (7.6%) and protospacer 47 (0.8%) in CRISPR 1 and CRISPR 2 loci, respectively, were domains of unknown function.

## 4. Discussion

Mastitis caused by *E. coli* is one of the most frequent diseases in dairy cattle resulting from environmental infection, and it is usually characterized by changes in milk composition and quality [1,2]. Although the relationship between virulence factors on bovine mastitis caused by *E. coli* and its clinical severity has not been fully elucidated, many studies have reported the influence of virulence factors on the establishment of clinically severe infections [6,31]. In this study, 18 out of 33 virulence genes associated with ExPEC were detected, and 89.6% of isolates from normal bulk tank milk carried one or more virulence genes. In particular, *fimH*, which is associated with the virulence factor adhesin, was detected the most often (80.9%). Guerra et al. (2020) [32] and Zhang et al. (2018) [33] also reported a 100% and 89.9% prevalence of *fimH*, respectively, indicating its ubiquity among mastitis-causing *E. coli* isolates. The *fimH* gene is a bacterial adhesin that helps *E. coli* bind to host cells and their receptors, and plays a crucial role in causing bovine mastitis by colonizing the mammary glands, resembling the pathogenesis of urinary tract infections [31,34,35]. Other virulence genes of adhesin, such as *afa/draBC*, *sfaS*, *bmaE*, and *papG allele 3*, were also detected in this study. The prevalence rates of these genes varied from 0.5% to 24.0%, but the presence of these adhesins also implies the facilitated attachment of bacteria onto host cells, helping the colonization of the region and increasing the possibility of mastitis [6,31].

Toxins encoded by virulence genes, such as *univcnf*, *cnf1*, *cdt*, *hlyA*, and *cdtB*, are considered essential in the pathogenesis of mastitis following colonization via adhesins. In this study, the most prevalent toxin gene was *univcnf* (21.9%), while the prevalence of other toxin genes ranged from 1.1% to 2.7%. Lehtolainen et al. (2003) [36] reported that cytotoxic necrotizing factors (CNF), encoded by *univcnf*, *cnf 1*, and *cnf 2*, are significantly associated with the persistence of mastitis. Moreover, the potential of CNFs to cause tissue damage or mediate bacteremia can lead to acute mastitis with severe systemic symptoms [37]. Therefore, if whole milk was derived from clinical mastitis rather than from a normal bulk tank, a higher prevalence may be confirmed.

Although the prevalence of the genes *iss* and *traT,* which are related to serum survival, was 38.3% and 26.8%, respectively, in this study, several reports have described the absence of a correlation between the presence of these genes and the pathogenicity of mastitis [31,38,39]. Therefore, it is difficult to predict whether the presence of *iss* and *traT* genes in *E. coli* from bulk tank milk may increase the risk of mastitis.

Phylogenetic analysis is increasingly being used as a modern method of determining virulence potential [40]. In this study, the phylogenetic group B1 (64.0%) was the most prevalent, followed by A (20.1%). On the other hand, phylogroups B2 and D, which were reported as highly virulent phylogroups regarding ExPEC [40,41], were detected at 0.0% and 8.5% prevalence, respectively. This result is in accordance with those of previous studies that phylogroups B1 and A were the most common groups in normal and mastitis milk samples, while phylogroups D and B2 were rarely detected [42]. According to previous studies, mastitis-causing *E. coli* isolates may be related to commensal isolates attaining virulence genes, causing infection in hosts with compromised immune systems [43,44].

*E. coli* contains four CRISPR loci: CRISPR 1, 2, 3 and 4; these are classified as either Type I-E (CRISPR 1 and 2) or Type I-F (CRISPR 3 and 4), depending on the presence of the associated *cas* genes [45]. In this study, 161 (98.2%) of 164 isolates possessing virulence genes were identified to possess CRISPR 1 and/or CRISPR 2, which comprise highly conserved direct repeat sequences with variable spacer sequences [22]. Meanwhile, CRISPR 3 and 4 loci, which possess a lower spacer distribution, were not detected. It was reported that CRISPR 1 and/or 2 loci have been preserved and stationary within *E. coli* over a long period [46], whereas CRISPR 3 and 4 loci are a recent creation [22]. Moreover, the hypervariability of CRISPR loci can be applied in phylogenetic analysis, as in previous reports [21,47]. In particular, Touchon et al. (2011) [22] reported that only the phylogenetic group B2 possessed CRISPR 4, implying that the absence of CRISPR 3 and 4 in this study could be linked to the absence of the phylogenetic group B2. Moreover, the absence of a significant difference in the distributions of CRISPR 1 and 2 in the phylogenetic groups C and D is also suggested to be linked with CRISPR loci and phylogeny.

Because both virulence genes and spacers of CRISPR are acquired by horizontal gene transfer via plasmids and phages [48,49], isolates with different virulence genes can have different distributions in CRISPR content, resulting in different ESTs. In this study, the distributions of EST 4 and EST 22 were significantly higher in isolates harboring *fimH*, *ompT*, *afa/draBC*, and *univcnf*, which play a crucial role in MPEC, compared to isolates carrying the genes *iss* and *traT*, which lack a role in pathogenicity. Therefore, these results suggest that the distribution of spacers may be reflected by the presence of virulence genes, as previously reported [24,50].

The CRISPR system of *E. coli* also functions as a regulatory mechanism and immune system of bacteria [22,51,52,53,54]. In this study, three (DNA-cytosine methyltransferase, DNA-binding protein, and helicase) of eight protospacers were associated with gene regulation. Bozic et al. (2019) [55] reported that CRISPR I-E (CRISPR 1 and 2) targets bacterial chromosomes, suggesting its major role in the regulation of endogenous genes. Moreover, protospacer 163, which is linked with bacteriophage tail-associated protein, is homologous to the Type VI secretion apparatus [56], which is associated with the increased virulence of many pathogens [57]. Interestingly, in this study, protospacer 163 was only detected in EST 4 and EST 22, which are ESTs with a significantly higher prevalence in isolates carrying four virulence genes (*fimH*, *ompT*, *afa/draBC*, and *univcnf*).

## 5. Conclusions

In conclusion, the results of protospacer distribution suggest that CRISPR I-E is linked with gene regulation and pathogenicity in *E. coli*. Moreover, the CRISPR sequence-typing approach helped to clarify and trace virulence potential, by showing significant differences in prevalence based on different virulence genes.

## Figures and Tables

**Table 1:** Distribution of virulence genes in *E. coli* isolated from bulk tank milk.

Virulence Genes ^1^	No (%) of Isolates Included by Company			
	A (*n* ^2^ = 38)	B (*n* = 42)	C (*n* = 103)	Total (*n* = 183)
*fimH*	36 (94.7) _a_	41 (100.0) _a_	71 (69.0) _b_	148 (80.9) ^A^
*iss*	27 (71.1) _a_	14 (34.1) _b_	29 (28.2) _b_	70 (38.3) ^B^
*traT*	21 (55.3) _a_	12 (29.3) _b_	16 (15.5) _b_	49 (26.8) ^B,C^
*ompT*	10 (26.3)	14 (34.1)	23 (22.3)	47 (25.7) ^B,C^
*afa/draBC*	6 (15.8)	11 (26.8)	27 (26.2)	44 (24.0) ^B,C^
*univcnf*	6 (15.8)	15 (36.6)	19 (18.4)	40 (21.9) ^B,C^
*iroN E. coli*	5 (13.2)	8 (19.5)	12 (11.7)	25 (13.7) ^C,D^
*kpsMT K5*	2 (5.3) _a,b_	7 (17.1) _a_	4 (3.9) _b_	13 (7.1) ^D,E^
*fyuA*	5 (13.2)	3 (7.3)	4 (3.9)	12 (6.6) ^D,E^
*sfaS*	3 (7.9)	1 (2.4)	8 (7.8)	12 (6.6) ^D,E^
*bmaE*	0 (0.0)	0 (0.0)	9 (8.7)	9 (4.9) ^D,E^
*cnf1*	2 (5.3)	0 (0.0)	3 (2.9)	5 (2.7) ^E^
*cdt*	0 (0.0)	1 (2.4)	3 (2.9)	4 (2.2) ^E^
*hlyA*	1 (2.6)	1 (2.4)	1 (1.0)	3 (1.6) ^E^
*cdtB*	0 (0.0)	0 (0.0)	2 (1.9)	2 (1.1) ^E^
*iutA*	1 (2.6)	0 (0.0)	0 (0.0)	1 (0.5) ^E^
*papG allele 3*	1 (2.6)	0 (0.0)	0 (0.0)	1 (0.5) ^E^
*kpsMT II*	1 (2.6)	0 (0.0)	0 (0.0)	1 (0.5) ^E^

^1^ PAI, cvaC, focG, ibeA, ireA, kpsMT K1, kpsMT III, nfaE, papAH, papC, papEF, papG allele 1, papG allele 2, rfc, and sfa/focDE genes were not detected in any of the isolates. ^2^ *n* = No. of *E. coli* isolated from each company. Values with different subscript letters represent significant differences among companies, while superscript letters represent significant differences in total (*p* < 0.05).

**Table 2:** Distribution of phylogenetic groups and CRISPR loci of 164 *E. coli* possessing virulence genes isolated from bulk tank milk.

Groups	No (%) of Isolates Included by Company			
	A (*n* ^1^ = 38)	B (*n* = 41)	C (*n* = 85)	Total (*n* = 164)
Phylogenetic groups				
A	3 (7.9) _b_	13 (31.7) _a_	17 (20.0) _a,b_	33 (20.1) ^B^
B1	26 (68.4)	20 (48.8)	59 (69.4)	105 (64.0) ^A^
B2	0 (0.0)	0 (0.0)	0 (0.0)	0 (0.0) ^D^
C	1 (2.6)	5 (12.2)	6 (7.1)	12 (7.3) ^C^
D	8 (21.1) _a_	3 (7.3) _a,b_	3 (3.5) _b_	14 (8.5) ^C^
E	0 (0.0)	0 (0.0)	0 (0.0)	0 (0.0) ^D^
F	0 (0.0)	0 (0.0)	0 (0.0)	0 (0.0) ^D^
CRISPR loci				
CRISPR 1	37 (97.4)	38 (92.7)	82 (96.5)	157 (95.7) ^A^
CRISPR 2	33 (86.8)	31 (75.6)	58 (68.2)	122 (74.4) ^B^
CRISPR 3	0 (0.0)	0 (0.0)	0 (0.0)	0 (0.0) ^C^
CRISPR 4	0 (0.0)	0 (0.0)	0 (0.0)	0 (0.0) ^C^

^1^ *n* = No. of *E. coli* isolated from each company. Values with different subscript letters represent significant differences among companies, while superscript letters represent significant differences in total (*p* < 0.05).

**Table 3:** CRISPR-based typing by virulence gene in 164 *E. coli* possessing virulence genes, isolated from bulk tank milk.

*E. coli* Sequence Types	No (%) of Isolates with Virulence Genes						
	*fimH* (*n* ^1^ = 148)	*iss* (*n* = 70)	*traT* (*n* = 49)	*ompT* (*n* = 47)	*afa/darBC* (*n* = 44)	*univcnf* (*n* = 40)	Total (*n* = 164)
EST 1	0 (0.0) _b_^B^	2 (2.9) _a,b_	0 (0.0) _b_	5 (10.6) _a,b_^A^	3 (6.8) _a,b_^B^	9 (22.5) _a_^A^	19 (11.6) ^C,D^
EST 2	2 (1.4) ^B^	1 (1.4)	0 (0.0)	1 (2.1) ^B^	0 (0.0) ^B^	1 (2.5) ^B^	5 (3.0) ^E,F^
EST 3	3 (2.0) ^B^	2 (2.9)	2 (4.1)	1 (2.1) ^B^	0 (0.0) ^B^	0 (0.0) ^B^	8 (4.9) ^E,F^
EST 4	16 (10.8) _a_^A^	2 (2.9) _b,c_	0 (0.0) _c_	6 (12.8) _a,b_^A^	9 (20.5) _a,b_^A^	5 (12.5) _a,b_^A^	38 (23.2) ^B^
EST 5	3 (2.0) ^B^	1 (1.4)	0 (0.0)	1 (2.1) ^B^	0 (0.0) ^B^	1 (2.5) ^B^	6 (3.7) ^E,F^
EST 6	2 (1.4) ^B^	0 (0.0)	0 (0.0)	0 (0.0) ^B^	0 (0.0) ^B^	0 (0.0) ^B^	2 (1.2) ^F^
EST 7	2 (1.4) ^B^	0 (0.0)	1 (2.0)	0 (0.0) ^B^	1 (2.3) ^B^	0 (0.0) ^B^	4 (2.4) ^F^
EST 8	3 (2.0) ^B^	2 (2.9)	3 (6.1)	2 (4.3) ^B^	2 (4.5) ^B^	0 (0.0) ^B^	12 (7.3) ^D,E^
EST 9	3 (2.0) ^B^	2 (2.9)	1 (2.0)	1 (2.1) ^B^	1 (2.3) ^B^	0 (0.0) ^B^	8 (4.9) ^E,F^
EST 10	7 (4.7) _a_^B^	4 (5.7) _a,b_	2 (4.1) _a,b_	3 (6.4) _a,b_^B^	0 (0.0) _b_^B^	2 (5.0) _a,b_^B^	18 (11.0) ^C,D^
EST 11	3 (2.0) ^B^	3 (4.3)	2 (4.1)	0 (0.0) ^B^	0 (0.0) ^B^	0 (0.0) ^B^	8 (4.9) ^E,F^
EST 12	1 (0.7) ^B^	0 (0.0)	1 (2.0)	0 (0.0) ^B^	1 (2.3) ^B^	0 (0.0) ^B^	3 (1.8) ^F^
EST 13	7 (4.7) _a_^B^	5 (7.1) _a,b_	1 (2.0) _a,b_	1 (2.1) _a,b_ ^B^	1 (2.3) _a,b_ ^B^	0 (0.0) _b_^B^	15 (9.1) ^D,E^
EST 14	8 (5.4) _a_^B^	6 (8.6) _a,b_	8 (16.3) _a_	0 (0.0) _b_^B^	2 (4.5) _a,b_^B^	1 (2.5) _a,b_^B^	25 (15.2) ^C^
EST 15	2 (1.4) ^B^	2 (2.9)	1 (2.0)	0 (0.0) ^B^	2 (4.5) ^B^	0 (0.0) ^B^	7 (4.3) ^E,F^
EST 16	8 (5.4) ^B^	7 (10.0)	8 (16.3)	5 (10.6) ^A^	4 (9.1) ^B^	1 (2.5) ^B^	33 (20.1) ^B^
EST 17	3 (2.0) ^B^	1 (1.4)	2 (4.1)	0 (0.0) ^B^	0 (0.0) ^B^	0 (0.0) ^B^	6 (3.7) ^E,F^
EST 18	3 (2.0) ^B^	0 (0.0)	0 (0.0)	0 (0.0) ^B^	0 (0.0) ^B^	0 (0.0) ^B^	3 (1.8) ^F^
EST 19	3 (2.0) ^B^	3 (4.3)	0 (0.0)	0 (0.0) ^B^	0 (0.0) ^B^	0 (0.0) ^B^	6 (3.7) ^E,F^
EST 20	3 (2.0) ^B^	0 (0.0)	3 (6.1)	0 (0.0) ^B^	0 (0.0) ^B^	0 (0.0) ^B^	6 (3.7) ^E,F^
EST 21	2 (1.4) ^B^	2 (2.9)	0 (0.0)	1 (2.1) ^B^	0 (0.0) ^B^	2 (5.0) ^B^	7 (4.3) ^E,F^
EST 22	33 (22.3) _a_^A^	9 (12.9) _b_	0 (0.0) _c_	10 (21.3) _b_^A^	13 (29.5) _b_^A^	9 (22.5) _b_^A^	74 (45.1) ^A^
EST 23	7 (4.7) _a_^B^	2 (2.9) _a,b_	4 (8.2) _a,b_	1 (2.1) _a,b_^B^	1 (2.3) _a,b_^B^	0 (0.0) _b_^B^	15 (9.1) ^D,E^
EST 24	10 (6.8) _a_^B^	7 (10.0) _a,b_	1 (2.0) _b_	5 (10.6) _a,b_^A^	0 (0.0) _b_^B^	7 (17.5) _a,b_^A^	30 (18.3) ^B,C^
EST 25	11 (7.4) _a_^A^	5 (7.1) _a,b_	8 (16.3) _a,b_	3 (6.4) _a,b_^B^	4 (9.1) _a,b_^B^	1 (2.5) _b_^B^	32 (19.5) ^B,C^
EST 26	3 (2.0) ^B^	2 (2.9)	1 (2.0)	1 (2.1) ^B^	0 (0.0) ^B^	1 (2.5) ^B^	8 (4.9) ^E,F^

^1^ *n* = No. of *E. coli* isolates harboring gene. Values with different subscript letters represent significant differences in the number of isolates in each EST, while values with different superscript letters represent significant differences in the number of isolates among ESTs (*p* < 0.05).

**Table 4:** Protospacers matching plasmids and phages, and spacer sequences in 164 *E. coli* possessing virulence genes, isolated from bulk tank milk.

CRISPR Array	Name of Protospacer	Sequences (5′ to 3′)	No. (%) of Isolates	Protospacer Match
CRISPR 1	1	ACATGAATGTCGGTTCAGACCGTGTTTTTACC	29 (18.5)	DNA-binding protein
		TGTACTTACAGCCAAGTCTGGCACAAAAATGG		
	78	CCCTCACACCGATTCGCCAAACGGTGGAGAAG	1 (0.6)	toll/interleukin-1 receptor domain-containing protein
		GGGAGTGTGGCTAAGCGGTTTGCCACCTCTTC		
	81	TTTTGCTGACACCGGCAATACTGAACGGCTGG	11 (7.0)	DNA-cytosine methyltransferase
		AAAACGACTGTGGCCGTTATGACTTGCCGACC		
	107	GCTGGTGGCGCGGGCAAACGGAACAATCCCGC	1 (0.6)	*darB*, helicase
		CGACCACCGCGCCCGTTTGCCTTGTTAGGGCG		
	117	AAACAGATTGTTCGTTTTCCCCATATTCATGA	12 (7.6)	DUF1380 domain-containing protein
		TTTGTCTAACAAGCAAAAGGGGTATAAGTACT		
	162	AGTATTAACTGCGGTGGCAGTGAGGCCAATAG	1 (0.6)	Head decoration protein, Viral protein
		TCATAATTGACGCCACCGTCACTCCGGTTATC		
	163	GTTGCCCCCCAAAATCATTAAATCCCCGGCGG	32 (20.4)	tail associated lysozyme
		CAACGGGGGGTTTTAGTAATTTAGGGGCCGCC		
CRISPR 2	47	GAAAAATGCATACGATTCGAGCACCAGTTTGGC	1 (0.8)	DUF1281 domain-containing protein
		CTTTTTACGTATGCTAAGCTCGTGGTCAAACCG		

## Data Availability

Not applicable.

## Supplementary Materials

The following supporting information can be downloaded at: https://www.mdpi.com/article/10.3390/ani12040503/s1, Figure S1: Grouping of 164 isolates into Escherichia sequence types (ESTs) according to spacer contents; Table S1: Names and sequences of all spacers in each CRISPR locus identified in this study.
